# Memory loss during lenalidomide treatment: a report on two cases

**DOI:** 10.1186/2050-6511-14-41

**Published:** 2013-08-12

**Authors:** Adeline Rollin-Sillaire, Xavier Delbeuck, Marianne Pollet, Marie-Anne Mackowiak, Pierre Lenfant, Marie-Pierre Noel, Thierry Facon, Xavier Leleu, Florence Pasquier, Emilie Le Rhun

**Affiliations:** 1 Univ Lille Nord de France, UDSL, Lille F-59000, France; 2 EA 1046, Excellence Laboratory DISTALZ, Memory Clinic, Lille University Hospital, Lille F-59000, France; 3 EA 1046, Department of Nuclear Medicine, F-59000 Lille, France; 4 Neurooncology, University Hospital, F59037 Lille, France; 5 Department of Medical Oncology, Oscar Lambret Center, F-59020 Lille, France

**Keywords:** Lenalidomide, Chemo brain, Chemo fog, Cognitive impairment, Episodic memory, Dementia

## Abstract

**Background:**

There are many reports of cognitive dysfunction in patients receiving chemotherapy or targeted therapies. Many antineoplastic agents may be involved in the condition also known as “chemo brain” or “chemo fog”.

**Case presentation:**

Two male patients (aged 41 and 70) with multiple myeloma developed severe, rapidly progressing cognitive impairment (mostly involving episodic memory) and loss of independence in activities of daily living during lenalidomide-based treatment. On withdrawal of the drug, one patient recovered normal cognitive function and independence in activities of daily living, whereas mild cognitive impairment persisted in the other patient. The Naranjo Adverse Drug Reaction Probability Scale score was 6 out of 13 for the first patient and 5 out of 13 for the second, suggesting a probable causal relationship between the adverse event and lenalidomide administration.

**Conclusion:**

Lenalidomide may induce particular cognitive disorders (notably episodic memory impairments) in some patients. The drug’s putative neurotoxicity is probably promoted by specific risk factors (such as previous chemotherapy, prior mild cognitive impairment, age and the presence of cerebrovascular lesions).

## Background

Several studies have suggested that both malignant tumors and their treatments (such as chemotherapy, targeted therapy and radiotherapy) may induce cognitive impairment [1-4]. Indeed, the terms “chemo brain” and “chemo fog” are often used to describe cognitive impairment observed before, during or after chemotherapy or systemic administration of other antineoplastic agents. Disabling cognitive disorders may affect between 15% and 50% of patients undergoing chemotherapy [5]. However, literature reports diverge as to the types and durations of cognitive impairments observed during chemotherapy. In most cases, the manifestations of chemo brain are mild but may have a slight impact on activities of daily living (ADL). The most common reported symptoms are fatigue, feelings of confusion or mental fogginess, inability to focus or concentrate, decreased attention span, slower thinking and impairments in remembering details, handling multiple tasks or learning new skills. Although a range of cognitive adverse effects have been observed during chemotherapy, impairments in memory, processing speed and executive function appear to be the most frequently reported [6,7]. The duration of the symptoms varies and long-term cognitive impairment has been observed [5]. Although most of the studies in this field concern cognitive complaints in women receiving adjuvant chemotherapy for breast cancer, cognitive complaints can occur in many cancer settings. Atrophy in some brain areas on magnetic resonance imaging (MRI) may be correlated with attention or visual memory performances [8,9].

Preliminary studies have suggested the existence of host-related risk factors (e.g. age, genetic polymorphisms, immune reactivity, nutritional factors, hormone profile and the lack of cognitive reserve) and disease-related factors (e.g. tumor gene mutations, the induction of pro-inflammatory cytokines and paraneoplastic disorders) that contribute to cognitive decline [10-12]. Little is known about the mechanism(s) of chemo brain, although several hypotheses have been put forward: direct neurotoxic effects, oxidative stress and DNA damage, induced hormonal changes, immune dysregulation, cytokine release, vascular injury and blood clotting in small vessels and a genetic predisposition [11,12]. There are number of guidelines and consensus statements on the assessment of cognitive function in chemotherapy trials [5,12-14].

Here, we report on two multiple myeloma (MM) patients who presented with memory impairments and reduced independence in ADL during treatment with lenalidomide.

## Case presentations

### Patient 1

The patient was a 41-year-old male with a history of arterial hypertension and aortic dissection. Two years previously, the patient had presented with an International Staging System (ISS) grade III immunoglobulin (Ig) G lambda MM. There was no chromosome 17 deletion or 4;14 translocation. The MM was initially treated with a three-month course of bortezomib and dexamethasone (as part of the induction phase with a view to possible transplantation) but merely stabilized. The patient then received vincristine/adriamycin/dexamethasone (VAD) as a salvage induction regimen that was subsequently withdrawn because of severe neuropathy. Treatment with a cyclophosphamide/adriamycin/dexamethasone (CAD) regimen was followed by a hematopoietic stem cell autograft for consolidation three months after initiation of the VAD regimen. At the age of 40 (i.e. after completion of the CAD regimen and after the autograft), the patient complained of memory loss and attention decline. A neuropsychological examination was first performed 13 months after the onset of the complaints and revealed moderately severe attention and executive disorders (with a score of 17 out of 30 in the Montreal Cognitive Assessment (MoCA) [15]). However, the patient also displayed mild language and episodic memory impairments. In a free and cued selective reminding test (FCSRT) [16,17], free recall was most strongly impaired (see Table 1). Brain MRI revealed a few white matter lesions and two microbleeds. We made a diagnosis of mild cognitive impairment due to chemo brain.

**Table 1:** Performance of Patient 1 in the free and cued selective reminding test (FCSRT) during follow-up

	**FCSRT free recall**	**FCSRT total recall (free and cued recall)**	**FCSRT delayed free recall**	**FCSRT delayed total recall**
Before lenalidomide treatment	20/48	40/48	6/16	13/16
During lenalidomide treatment	10/48	25/48	6/16	12/16
After lenalidomide withdrawal	31/48	45/48	12/16	13/16
1 year after lenalidomide withdrawal	27/48	43/48	13/16	16/16

**Figure 1:**
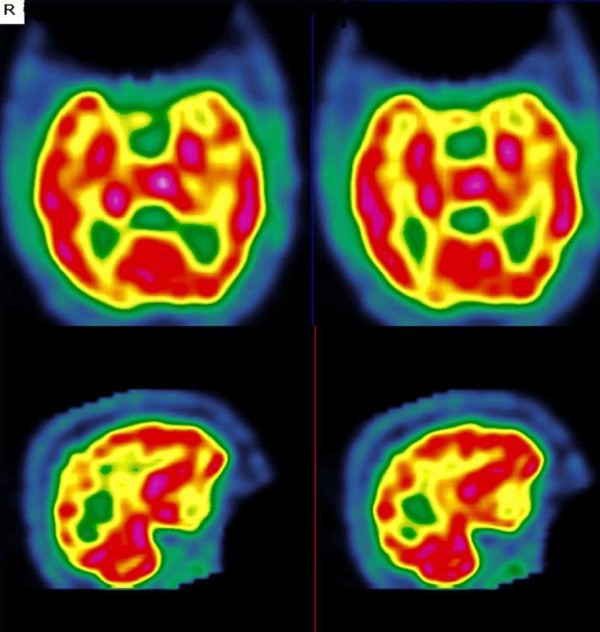
**Results from the first SPECT scan (during the course of lenalidomide treatment).** Note the left temporal hypoperfusion in the transverse (top) and sagittal (bottom) slices.

Eighteen months after the hematopoietic stem cell autograft, bone pain and an increase in the serum free light lambda chain level (1250 mg/L in a Freelite® assay) prompted the introduction of combination therapy with lenalidomide and dexamethasone in a first relapse setting. One month later, the patient’s cognitive impairment progressively but significantly worsened and had a marked impact on ADL. Two months after the initiation of lenalidomide/ dexamethasone therapy, the patient was admitted to our university medical center for cognitive assessment. A neurological examination revealed psychomotor impairment, lower limb sensory ataxia and the abolition of reflexes. In a second cognitive assessment, the patient was found to have a MoCA score of 20 out of 30 and an overall decrease in episodic memory performance (in both free and cued recall) (see Table 1). We then sought to establish the etiology of this cognitive impairment. On the basis of the Freelite® test results, the patient had a very good partial response. Serological tests for paraneoplastic antibodies, HIV, syphilis, hepatitis, Lyme’s disease, Whipple disease and autoimmune diseases were negative. Assay results for metabolic disease (plasma and urinary copper, plasma very long chain fatty acids and blood amino acid, lactate and pyruvate levels) and thyroid function were normal. The only finding was a low thiamine level (64nmol/L, compared with a normative value of >95nmol/L). No fragile X mutation was found. Examination of the cerebrospinal fluid (CSF) confirmed the absence of malignant cells and oligoclonal bands. Proteinorachia and glycorachia values were normal. Cerebrospinal fluid bacterial cultures, neurotropic virus cultures and tuberculosis mycobacterium polymerase chain reaction assays were all negative. The patient tested negative for 14.3.3 protein. Although the brain MRI results had not changed since the previous scan, single photon emission computed tomography (SPECT) with 99m Tc-HMPAO revealed left temporal hypoperfusion (Figure 1). Electroencephalography showed slow activity. The ophthalmological examination was normal. An electromyogram revealed chemotherapy-induced sensorimotor axonal neuropathy. Nevertheless, the rapid cognitive decline was still unexplained on release of the patient from hospital.

**Figure 2:**
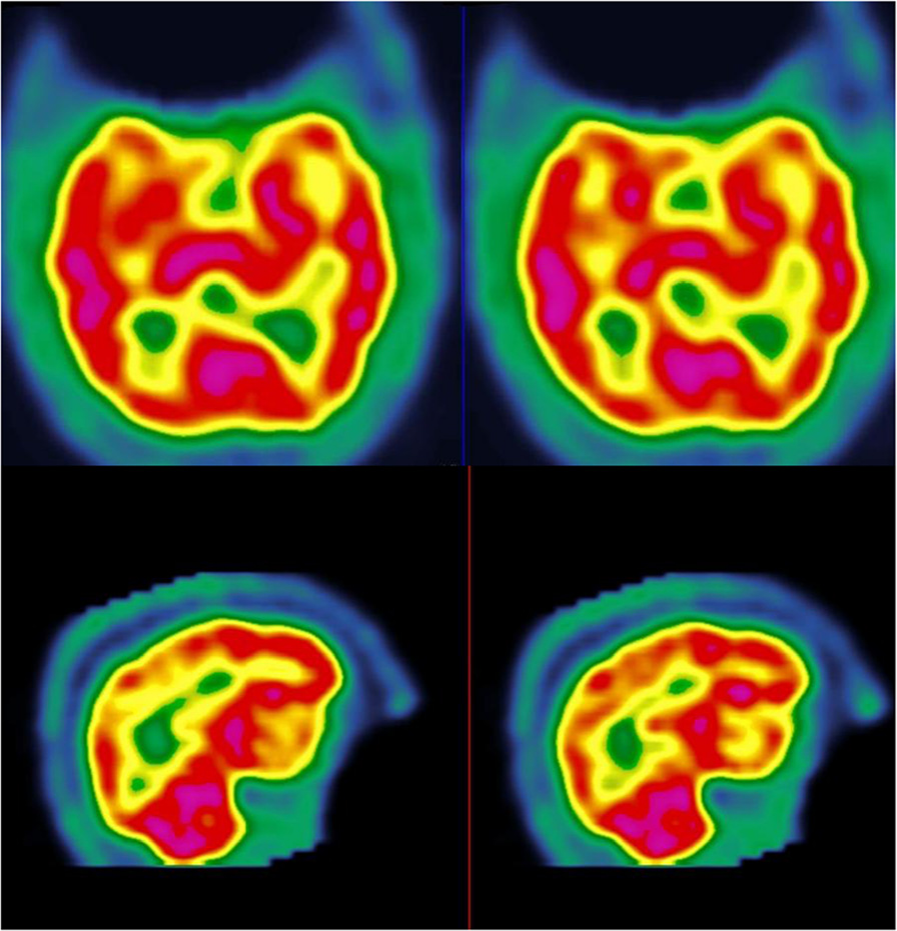
**Results from the second SPECT (after the cessation of lenalidomide treatment).** Note the normal perfusion in the transverse (top) and sagittal (bottom) slices.

One month later, the patient’s state worsened, with the development of marked apathy, anorexia and somnolence. A clinical examination revealed a sacral pressure ulcer. Laboratory tests evidenced pancytopenia (platelet count: 46 ×10^9^/L; neutrophil count: 0.2 ×10^9^/L; hemoglobin level: 7.6g/dl) and an inflammatory syndrome (C-reactive protein: 175mg/L). The brain MRI, electroencephalography and CSF assay results were unchanged. After hospitalization, the patient developed fever with diarrhea caused by non-typhi *Salmonella* (prompting the initiation of antibiotic treatment). Given the severe neutropenia and the infectious syndrome, lenalidomide was withdrawn and replaced by cyclophosphamide. The patient’s cognitive functions were seen to have improved during hospitalization, with a Mini Mental State Examination (MMSE) score [18] of 28 out of 30 one month after the withdrawal of lenalidomide. Three months later, the MoCA score was 27 out of 30. Cognitive function (and particularly episodic memory performance) had improved (Table 1). One year later, the cognitive profile had stabilized (with the persistence of mild attention disorders) and the 99m Tc-HMPAO SPECT results were normal (Figure 2).

### Patient 2

The second patient was a 70 year-old male admitted to our hospital for progressive cognitive decline. He had a history of ISS stage I IgG kappa MM (in the absence of chromosome 17 deletion or a 4;14 translocation) with onset six years previously. He had initially been treated with two VAD induction cycles and a subsequent hematopoietic stem cell autograft and had shown a partial response. After three treatment-free years, the patient presented with a progression of MM. Combination therapy with bortezomib and an anti-insulin-like growth factor-1 antibody was introduced. These agents were withdrawn after 4 months because of the occurrence of severe neuropathy and were not replaced. Eighteen months later, combination therapy with lenalidomide, dexamethasone and an anti-CS1 antibody was initiated following an increase in the M component, with no signs of hyperviscosity syndrome. After one month of treatment, the patient complained of memory loss, apathy and word-finding difficulties, with repercussions on ADL. An initial cognitive assessment revealed a severe impairment in episodic memory (with poor free and cued recall in the FCSRT, see Table 2), whereas other cognitive functions (such as executive function and construction) were within the normal range. The patient’s global cognitive efficiency was scored as 128 out of 144 on the Mattis Dementia Rating Scale (DRS) [19] and 26 out of 30 in the MMSE. The neurological examination was normal. Brain MRI revealed widespread white matter lesions and overall atrophy. The patient was offered regular clinical monitoring. Seven months later, the patient developed erythroderma and so lenalidomide was withdrawn. The patient and his wife both reported an overall improvement in cognitive function, with a decrease in apathy and improved memory. The patient had recovered his independence in ADL. A cognitive assessment was performed six months after the withdrawal of lenalidomide treatment. Although the patient’s global cognitive efficiency was stable (128 out of 144 on the Mattis DRS and 27 out of 30 in the MMSE), a slight improvement in performance in the FCSRT was apparent (see Table 2). Given that episodic memory was still the main dysfunction in our patient, we made a diagnosis of amnesic mild cognitive impairment. In an evaluation 12 months later, performance in the FCSRT was still improved.

**Table 2:** Performance of Patient 2 in the free and cued selective reminding test (FCSRT) during follow-up

	**FCSRT free recall**	**FCSRT total recall (free and cued recall)**	**FCSRT delayed free recall**	**FCSRT delayed total recall**
During lenalidomide treatment	6/48	29/48	1/16	10/16
6 months after lenalidomide withdrawal	12/48	35/48	4/16	12/16
12 months after lenalidomide withdrawal	11/48	37/48	3/16	13/16

## Discussion

Here, we described two patients in whom cognitive impairments developed or worsened significantly during lenalidomide treatment. In both cases, the neuropsychological manifestations and the impact on ADL appeared within one month of lenalidomide initiation. Both patients presented with a severe impairment in episodic memory, i.e. the ability to learn and retain new information. This impairment was evidenced in both cases by poor performance in the FCSRT. Moreover, the patients showed also evidence of a significant impairment in social and occupational functioning.

Lenalidomide is an immunomodulatory derivative of thalidomide that is approved for the treatment of first relapse in MM and is currently in clinical development for other malignant hematologic indications. The drug has anti-tumor, anti-angiogenesis and immunomodulatory effects. The most frequently reported adverse event (AE) for lenalidomide is immunosuppression (mainly neutropenia and thrombocytopenia), although the drug is considered as having a predictable safety profile and manageable effects. Venous thromboembolism is the most frequent non-hematologic AE [20]. In contrast to thalidomide, lenalidomide is not associated with a significant level of peripheral neurotoxicity. The incidence of grade 3 or 4 peripheral neuropathy reported in Phase III trials is below 2% and lenalidomide does not appear to worsen preexisting peripheral neuropathy [20,21]. A death in a Phase III study of 176 lenalidomide-treated MM patients was ascribed to leukoencephalopathy but no details were provided [20]. However, we are not aware of any reports of major cognitive impairments and ADL difficulties associated with lenalidomide administration.

Several lines of evidence suggest that lenalidomide was responsible for the cognitive impairment observed in our two patients. Firstly, there was a temporal relationship between the observed disorders and lenalidomide administration. Even though Patient 1 complained of cognitive impairment before starting lenalidomide, his episodic memory clearly worsened after lenalidomide was administered. This worsening was very acute and unexpected. Despite wide-ranging investigations (MRI, lumbar puncture and laboratory tests), no infectious, vascular, metabolic or paraneoplastic etiologies were found. In the second case, neither the patient nor his wife reported the presence of cognitive difficulties prior to initiation of lenalidomide. The cognitive disorders occurred within one months of the start of lenalidomide treatment. Secondly, we noticed a significant improvement in episodic memory (a major improvement for the first patient and a slight improvement for the second) and independence in ADL after lenalidomide withdrawal. This improvement in memory performance was still present a year later in both cases. Thirdly, the presence of left temporal hypoperfusion on SPECT fits with the observed impairment in the FCSRT. When lenalidomide was withdrawn, the SPECT results normalized. These changes may indicate that lenalidomide has a toxic effect on temporal structures. To the best of our knowledge, this is the first report of brain hypoperfusion associated with lenalidomide treatment.

Nevertheless, it is not possible to unequivocally state that lenalidomide is directly neurotoxic. Indeed, the Naranjo Adverse Drug Reaction Probability score was 6 out of 13 for Patient 1 and 5 out 13 for Patient 2, which corresponds to a probable adverse drug reaction [22]. A score above 9 corresponds to a definite adverse drug reaction, which was not the case here – primarily because (i) there are no previous conclusive reports on this reaction and (ii) the drug was not detected in blood (or other fluids) in concentrations known to be toxic. However, both patients had several putative risk factors for chemo brain, which may well explain their very poor cognitive tolerance of lenalidomide. Both had already undergone a cycle of chemotherapy before starting on lenalidomide and Patient 1 had already complained of cognitive impairment. The various drugs used to treat Patient 1’s MM (bortezomib, vincristine, dexamethasone and especially adriamycin [23,24]) could have been involved in chemo brain. However, Patient 2 did not have any memory complaints prior to lenalidomide initiation – despite having already received several antineoplastic agents. Patient 2 was elderly; cognitive assessment after lenalidomide withdrawal revealed the persistence of an episodic memory impairment in the FCSRT, which is a predictive factor for prodromal Alzheimer’s disease (AD) [25]. Moreover, MRI showed overall atrophy of the brain (including the hippocampal regions) and many white matter lesions. Thus, a diagnosis of prodromal AD could be considered in this case. Cerebrospinal fluid analysis with AD biomarker assays would have refined this diagnosis [26]. The presence of cerebrovascular lesions can also increase the risk of cognitive impairment. These observations suggest the existence of risk factors for chemo brain. Indeed, it has been suggested that age, lack of cognitive reserve, genetic risk factors, comorbid conditions and other cancer-related symptoms contribute to chemo brain [10]. Moreover, the results of longitudinal studies have shown that 20% to 30% of patients have cognitive deficits prior to the start of treatment [5,27-29]. Some researchers have assessed cognitive functions immediately after cancer diagnosis and prior to starting chemotherapy (which is an especially stressful period) and have suggested that cognitive deficits may be stress-related [27]. The presence of the apolipoprotein E ϵ4 (APOE E4) allele (which constitutes a strong risk factor for AD) [30] has also been suggest as a risk factor for chemo brain. A study found that E4 allele carriers being treated for breast cancer or lymphoma with chemotherapy tended to score less well in tests of visual memory, spatial ability and psychomotor functioning than survivors with other apolipoprotein alleles [10]. The potential mechanism of the interaction between chemotherapy and APOE status remains unclear. We did not perform APOE genotyping here because it is not currently recommended in the diagnosis of AD or other forms of dementia [31].

The putative mechanism by which lenalidomide may have an impact on cognition is also unknown. Lenalidomide has anti-angiogenic, anti-inflammatory and anti-neoplastic effects in preclinical models [32]. An anti-angiogenic effect might prohibit neurogenesis in hippocampal structures. Furthermore, it is not known to what extent lenalidomide can cross the blood–brain barrier (BBB) into the central nervous system (CNS). There are two reports of the remission of CNS progressions of hematologic malignancies (one diffuse large B cell lymphoma and one blastic mantle cell lymphoma) after lenalidomide treatment [33,34]; this suggests that lenalidomide may be able to cross a damaged BBB. We suspect that lenalidomide crossed the BBN in our two cases reports, albeit in the absence of a clearly identified mechanism. As with chemo brain in general, the mechanism of lenalidomide’s putative neurotoxicity is not clear. Several mechanisms could be suspected: direct neurotoxic effect of the drug, oxidative stress and DNA damage, induced hormonal changes, immune dysregulation, cytokine release, vascular injury and blood clotting in small vessels and a genetic predisposition.

## Conclusion

Here, we reported on the potential negative impact of lenalidomide on episodic memory in two MM patients undergoing chemotherapy. Although the symptoms were reversible, they were severe enough to lead to a loss of independence in ADL. The mechanism of these specific cognitive deficits is unknown. Lenalidomide’s putative neurotoxicity is probably exacerbated by risk factors (e.g. previous mild cognitive impairment, prior chemotherapy, age, cerebrovascular lesions, etc.) and some patients may be particularly vulnerable to the onset of cognitive impairments and a subsequent loss of independence in ADL.

There is a need to further identify and characterize the risks of cognitive impairment in patients being treated with immunomodulators.

### Consent

Written informed consent was obtained from both patients for publication of this case report and the accompanying images. A copy of the written consent form is available for review by the Editor-in-Chief of this journal.

## Abbreviations

ADL: Activities of daily living; MRI: Magnetic resonance imaging; DNA: Deoxyribo nucleic acid; MM: Multiple myeloma; Ig: immunoglobulin; ISS: International staging system; VAD: Vincristine/adriamycine/dexamethasone; CAD: Cyclophosphamide/adriamycine/dexamethasone; MoCA: Montreal cognitive assessment; FCSRT: Free and cued selective reminding test; CSF: Cerebro spinal fluid; SPECT: Single photon emission computed tomography; MMSE: Mini mental state examination; DRS: Dementia rating scale; AE: Adverse event; AD: Alzheimer’s disease; APOE: APOlipoprotein E; BBB: Blood brain barrier; CNS: Central nervous system.

## Competing interests

XL received grant support, lecture fees and board honoraria from Celgene. TF received lecture fees and board honoraria from Celgene. The others authors declare that they have no competing interests.

## Authors’ contributions

ARS provided patient care and wrote the draft. XD and MP performed neuropsychological examination and revised the draft. MAM, MPN, XL provided patient care and revised the draft. PL performed SPECT imaging and revised the SPECT examination. TF revised the draft. FP revised the draft and has given final approval of the version to be published. EL provided patient care, revised the draft and has given final approval of the version to be published. All authors have read and approved the final manuscript.

## Pre-publication history

The pre-publication history for this paper can be accessed here:

http://www.biomedcentral.com/2050-6511/14/41/prepub

## References

[B1] Anderson-Hanley C Sherman ML Riggs R Agocha VB Compas BE Neuropsychological effects of treatments for adults with cancer: a meta-analysis and review of the literature J Int Neuropsychol Soc 2003 9 967 982 14738279 10.1017/S1355617703970019

[B2] Jansen CE Miaskowski C Dodd M Dowling G Kramer J A metaanalysis of studies of the effects of cancer chemotherapy on various domains of cognitive function Cancer 2005 104 2222 2233 10.1002/cncr.21469 16206292

[B3] Stewart A Bielajew C Collins B Parkinson M Tomiak E A meta-analysis of the neuropsychological effects of adjuvant chemotherapy treatment in women treated for breast cancer Clin Neuropsychol 2006 20 76 89 10.1080/138540491005875 16410227

[B4] Falleti MG Sanfilippo A Maruff P Weih L Phillips K-A The nature and severity of cognitive impairment associated with adjuvant chemotherapy in women with breast cancer: a meta-analysis of the current literature Brain Cogn 2005 59 60 70 10.1016/j.bandc.2005.05.001 15975700

[B5] Wefel JS Vardy J Ahles T Schagen SB International cognition and cancer task force recommendations to harmonise studies of cognitive function in patients with cancer Lancet Oncol 2011 12 703 708 10.1016/S1470-2045(10)70294-1 21354373

[B6] Wefel JS Witgert ME Meyers CA Neuropsychological sequelae of non-central nervous system cancer and cancer therapy Neuropsychol Rev 2008 18 121 131 10.1007/s11065-008-9058-x 18415683

[B7] Correa DD Ahles TA Neurocognitive changes in cancer survivors Cancer J 2008 14 396 400 10.1097/PPO.0b013e31818d8769 19060604

[B8] McDonald BC Conroy SK Ahles TA West JD Saykin AJ Gray matter reduction associated with systemic chemotherapy for breast cancer: a prospective MRI study Breast Cancer Res Treat 2010 123 819 828 10.1007/s10549-010-1088-4 20690040 PMC3661415

[B9] Inagaki M Yoshikawa E Matsuoka Y Sugawara Y Nakano T Akechi T Wada N Imoto S Murakami K Uchitomi Y Smaller regional volumes of brain gray and white matter demonstrated in breast cancer survivors exposed to adjuvant chemotherapy Cancer 2007 109 146 156 10.1002/cncr.22368 17131349

[B10] Ahles TA Saykin AJ Noll WW Furstenberg CT Guerin S Cole B Mott LA The relationship of APOE genotype to neuropsychological performance in long-term cancer survivors treated with standard dose chemotherapy Psychooncology 2003 12 612 619 10.1002/pon.742 12923801

[B11] Ahles TA Saykin AJ Candidate mechanisms for chemotherapy-induced cognitive changes Nat Rev Cancer 2007 7 192 201 10.1038/nrc2073 17318212 PMC3329763

[B12] Vardy J Wefel JS Ahles T Tannock IF Schagen SB Cancer and cancer-therapy related cognitive dysfunction: an international perspective from the Venice cognitive workshop Ann Oncol 2008 19 623 629 17974553 10.1093/annonc/mdm500

[B13] Vardy J Rourke S Tannock IF Evaluation of cognitive function associated with chemotherapy: a review of published studies and recommendations for future research J Clin Oncol 2007 25 2455 2463 10.1200/JCO.2006.08.1604 17485710

[B14] Tannock IF Ahles TA Ganz PA Van Dam FS Cognitive impairment associated with chemotherapy for cancer: report of a workshop J Clin Oncol 2004 22 2233 2239 10.1200/JCO.2004.08.094 15169812

[B15] Nasreddine ZS Phillips NA Bédirian V Charbonneau S Whitehead V Collin I Cummings JL Chertkow H The Montreal Cognitive Assessment, MoCA: a brief screening tool for mild cognitive impairment J Am Geriatr Soc 2005 53 695 699 10.1111/j.1532-5415.2005.53221.x 15817019

[B16] Van Der Linden M Coyette F Pitrenaud J GREMEM M Van Der Linden M, Adam S, Agniel A, GREMEM M L’épreuve de rappel libre/rappel indicé à 16 items (RL/RI-16) L’évaluation des troubles de la mémoire 2004 Marseille: Présentation de quatre tests de mémoire épisodique (avec leur étalonnage) 25 47

[B17] Grober E Buschke H Guenine memory deficits in dementia Dev Neuropsychol 1987 3 13 36 10.1080/87565648709540361

[B18] Folstein MF Folstein SE McHugh PR « Mini-mental state ». A practical method for grading the cognitive state of patients for the clinician J Psychiatr Res 1975 12 189 198 10.1016/0022-3956(75)90026-6 1202204

[B19] Mattis S Bellak L, Karasu T Mental status examination for organic mental syndrome in the elderly patients Geriatric psychiatry: a handbook for psychiatrists and primary care physicians 1973 New York: Grune and Stratton 77 101

[B20] Dimopoulos M Spencer A Attal M Prince HM Harousseau J-L Dmoszynska A San Miguel J Hellmann A Facon T Foà R Corso A Masliak Z Olesnyckyj M Yu Z Patin J Zeldis JB Knight RD Multiple Myeloma (010) Study Investigators Lenalidomide plus dexamethasone for relapsed or refractory multiple myeloma N Engl J Med 2007 357 2123 2132 10.1056/NEJMoa070594 18032762

[B21] Weber DM Chen C Niesvizky R Wang M Belch A Stadtmauer EA Siegel D Borrello I Rajkumar SV Chanan-Khan AA Lonial S Yu Z Patin J Olesnyckyj M Zeldis JB Knight RD Multiple Myeloma (009) Study Investigators Lenalidomide plus dexamethasone for relapsed multiple myeloma in North America N Engl J Med 2007 357 2133 2142 10.1056/NEJMoa070596 18032763

[B22] Naranjo CA Busto U Sellers EM Sandor P Ruiz I Roberts EA Janecek E Domecq C Greenblatt DJ A method for estimating the probability of adverse drug reactions Clin Pharmacol Ther 1981 30 239 245 10.1038/clpt.1981.154 7249508

[B23] Ahles TA Saykin AJ Breast cancer chemotherapy-related cognitive dysfunction Clin Breast Cancer 2002 3 Suppl 3 84 90 10.3816/cbc.2002.s.018 12533268

[B24] Ahles TA Saykin AJ Furstenberg CT Cole B Mott LA Skalla K Whedon MB Bivens S Mitchell T Greenberg ER Silberfarb PM Neuropsychologic impact of standard-dose systemic chemotherapy in long-term survivors of breast cancer and lymphoma J Clin Oncol 2002 20 485 493 10.1200/JCO.20.2.485 11786578

[B25] Sarazin M Berr C De Rotrou J Fabrigoule C Pasquier F Legrain S Michel B Puel M Volteau M Touchon J Verny M Dubois B Amnestic syndrome of the medial temporal type identifies prodromal AD: a longitudinal study Neurology 2007 69 1859 1867 10.1212/01.wnl.0000279336.36610.f7 17984454

[B26] Dubois B Feldman HH Jacova C Dekosky ST Barberger-Gateau P Cummings J Delacourte A Galasko D Gauthier S Jicha G Meguro K O'brien J Pasquier F Robert P Rossor M Salloway S Stern Y Visser PJ Scheltens P Research criteria for the diagnosis of Alzheimer’s disease: revising the NINCDS-ADRDA criteria Lancet Neurol 2007 6 734 746 10.1016/S1474-4422(07)70178-3 17616482

[B27] Hermelink K Untch M Lux MP Kreienberg R Beck T Bauerfeind I Münzel K Cognitive function during neoadjuvant chemotherapy for breast cancer: results of a prospective, multicenter, longitudinal study Cancer 2007 109 1905 1913 10.1002/cncr.22610 17351951

[B28] Wefel JS Lenzi R Theriault RL Davis RN Meyers CA The cognitive sequelae of standard-dose adjuvant chemotherapy in women with breast carcinoma: results of a prospective, randomized, longitudinal trial Cancer 2004 100 2292 2299 10.1002/cncr.20272 15160331

[B29] Quesnel C Savard J Ivers H Cognitive impairments associated with breast cancer treatments: results from a longitudinal study Breast Cancer Res Treat 2009 116 113 123 10.1007/s10549-008-0114-2 18629633

[B30] Bickeböller H Campion D Brice A Amouyel P Hannequin D Didierjean O Penet C Martin C Pérez-Tur J Michon A Dubois B Ledoze F Thomas-Anterion C Pasquier F Puel M Demonet JF Moreaud O Babron MC Meulien D Guez D Chartier-Harlin MC Frebourg T Agid Y Martinez M Clerget-Darpoux F Apolipoprotein E and Alzheimer disease: genotype-specific risks by age and sex Am J Hum Genet 1997 60 439 446 9012418 PMC1712413

[B31] Atkins ER Panegyres PK The clinical utility of gene testing for Alzheimer’s disease Neurol Int 2011 3 e1 21785673 10.4081/ni.2011.e1 PMC3141112

[B32] Cives M Milano A Dammacco F Silvestris F Lenalidomide in multiple myeloma: current experimental and clinical data Eur J Haematol 2012 88 279 291 10.1111/j.1600-0609.2011.01735.x 22085323

[B33] Rubenstein JL Treseler PA Stewart PJ Regression of refractory intraocular large B-cell lymphoma with lenalidomide monotherapy J Clin Oncol 2011 29 e595 e597 10.1200/JCO.2011.34.7252 21519022 PMC3675664

[B34] Cox MC Mannino G Lionetto L Naso V Simmaco M Spiriti MAA Lenalidomide for aggressive B-cell lymphoma involving the central nervous system? Am J Hematol 2011 86 957 10.1002/ajh.22148 21990093

